# *Kaempferia parviflora* Extract as a Potential Anti-Acne Agent with Anti-Inflammatory, Sebostatic and Anti-*Propionibacterium acnes* Activity

**DOI:** 10.3390/ijms19113457

**Published:** 2018-11-03

**Authors:** Solee Jin, Mi-Young Lee

**Affiliations:** 1 Department of Medical Science, College of Medical Science, SoonChunHyang University, 22 SoonChunHyang-ro, Asan, Chungnam 31538, Korea; thfdl3076@gmail.com; 2 Department of Medical Biotechnology, College of Medical Science, SoonChunHyang University, 22 SoonChunHyang-ro, Asan, Chungnam 31538, Korea

**Keywords:** *Kaempferia parviflora*, anti-inflammation, sebostatic effect, anti-*P. acnes* effect

## Abstract

*Kaempferia parviflora*, referred to as black ginger, has traditionally been used as a health-promoting alternative medicine. In this study, we examined the anti-inflammatory, sebostatic, and anti-*Propionibacterium acnes* activities of *K. parviflora* extract. The extract significantly down-regulated the expression of inducible NO synthase (iNOS) and cyclooxygenase-2 (COX-2), and pro-inflammatory cytokine tumor necrosis factor alpha (TNF-α) level. Moreover, the phosphorylation of IĸBα and nuclear factor-kappa B (NF-κB), and the enhanced nuclear translocation of NF-κB p65 in lipopolysaccharide-stimulated murine macrophage-like cell line (RAW 264.7) cells were markedly decreased by the extract. Notably, the main component of *K. parviflora*, 5,7-dimethoxyflavone, also modulated the expression of iNOS and NF-κB signal molecules in *P. acnes*-stimulated human keratinocyte (HaCaT) cells. Additionally, *K. parviflora* extract inhibited the lipogenesis of sebocytes, as evidenced by a reduced level of triglyceride and lipid accumulation in the sebocytes. The sebostatic effect was also confirmed by a reduced expression of peroxisome proliferation-activating receptors (PPAR-γ) and oil-red O staining in sebocytes. Taken together, this study suggests for the first time that *K. parviflora* extract could be developed as a potential natural anti-acne agent with anti-inflammatory, sebostatic, and anti-*P. acnes* activity.

## 1. Introduction

Acne vulgaris is one of the most common dermatological diseases, affecting 80–85% of teenagers globally. It is triggered by several skin flora, including *Propionibacterium acnes* and *Staphylococcus aureus* [1,2,3]. The pathogenesis of acne includes induction of inflammatory responses, increased production of sebum, and hyperplasia of sebaceous glands, as well as changes in the lipid composition of sebum via lipogenesis modulation [4,5]. In addition, the level of linoleic acid (LA) in sebum and the sphingolipid level in the stratum corneum of acne patients was reported to be lower than that of people with no acne [6].

One of the underlying pathogenic mechanisms specifically involved in acne pathogenesis has been evident through an elevated expression of cyclooxygenase-2 (COX-2) and prostaglandin E2 (PGE_2_) associated with an enhanced release of pro-inflammatory cytokines and lipogenesis in sebocytes. Inflammatory response in aggravated and augmented acne lesions further underlines the role of peroxisome proliferation-activating receptors (PPARs), along with insulin and an insulin-like growth factor (IGF-1) [7]. Moreover, the interplay between lipid signals and inflammatory responses has been suggested as a cause of acne development. Therefore, finding new targets within the inflammatory and sebostatic response associated with sebum deregulation might be an innovative therapeutic strategy for acne.

Acne treatment with synthetic chemical medicines, such as antibiotics and steroids, can result in mild to severe side effects [8]. Thus, several complementary and alternative medicines, such as herbal extracts, plant oils, and antimicrobial peptides, have been used with far fewer side effects. However, the limited scientific and clinical data on the efficacy of these remedies are concerning, and they need to be complemented by further research.

*Kaempferia parviflora*, also known as black ginger or “krachai dum” in Thai, is a herbaceous plant belonging to the Zingiberaceae family [9]. It has been traditionally used as a health-promoting alternative medicine with anti-inflammatory, anti-allergic, anticholinesterase, adaptogenic, and anti-obesity effects [10]. *K. parviflora* contains several flavonoids, including 5,7-dimethoxyflavone, 5-hydroxy-3,7,4′-trimethoxyflavone, and 5-hydroxy-3,7-dimethoxyflavone [11]. The extracts of this plant have shown efficacies against several disorders, including metabolic, sexual, and cognitive disorders, as well as cancer [12]. However, the specific efficacy of *K. parviflora* extracts on acne vulgaris remains to be discovered. The present study suggested for the first time that *K. parviflora* ethanolic extracts might contribute to the amelioration of acne vulgaris by modulating the growth of *P. acnes*, sebocyte lipogenesis, and the inflammatory response.

## 2. Results

### 2.1. Anti-P. acnes Activity of K. parviflora Extract

**Figure 1:**
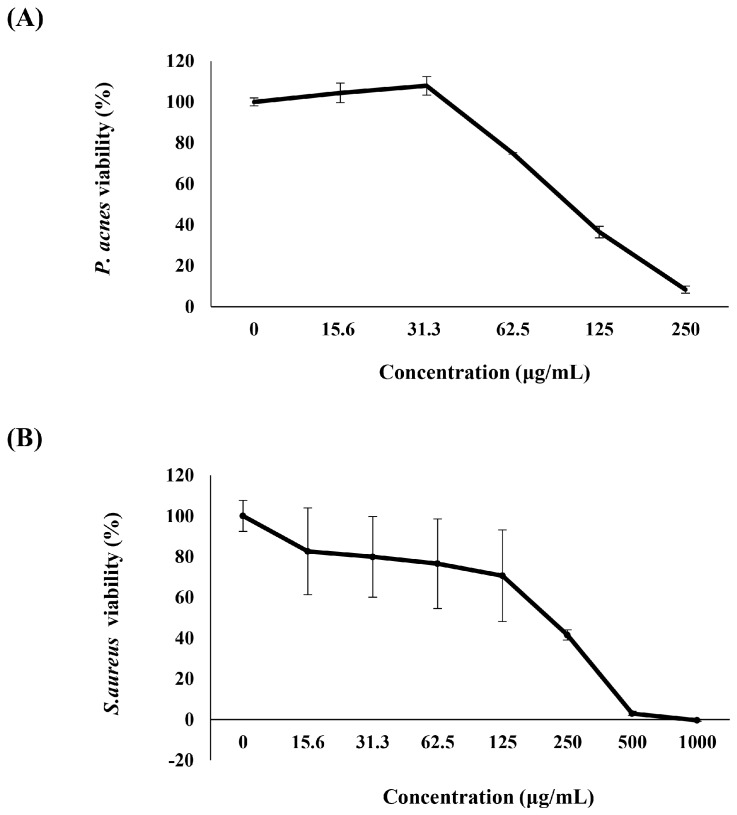
Antimicrobial effect of *Kaempferia parviflora* extract against (**A**) *Propionibacterium acnes* and (**B**) *Staphylococcus aureus*. All data are presented as the mean ± standard deviation (SD) of three independent experiments.

The antimicrobial effect of *K. parviflora* extract was investigated against two skin floras, *P. acnes* and *S. aureus*. As shown in Figure 1, 250 and 500 μg/mL of *K. parviflora* extract induced almost complete inactivation of *P. acnes* and *S. aureus*, respectively. These results suggested that *K. parviflora* extract possessed anti-acne properties, activated via inhibiting the growth of skin bacteria including *P. acnes* and *S. aureus*.

### 2.2. Anti-Inflammatory Effect of K. parviflora Extract

**Figure 2:**
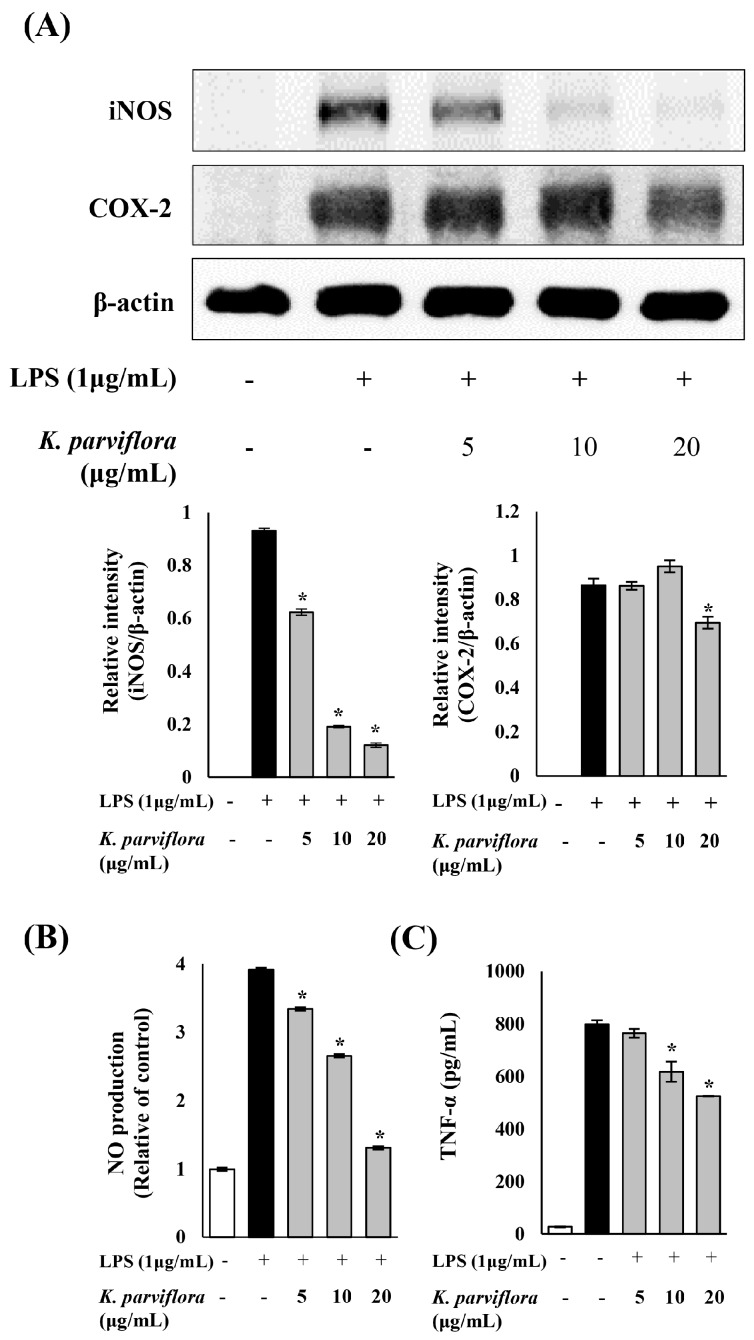
(**A**) Inhibitory effect of the *Kaempferia parviflora* extract on the expression of lipopolysaccharide (LPS)-induced inflammatory proteins, inducible NO synthase (iNOS) and cyclooxygenase-2 (COX-2), in murine macrophage-like cell line (RAW 264.7) cells. The expressions of iNOS and COX-2 were analyzed with ImageJ and normalized against β-actin; (**B**) effect of *K. parviflora* extract on NO production in LPS-induced inflammation in RAW 264.7 cells; (**C**) inhibitory effect of *K. parviflora* extract on LPS-induced TNF-α level in RAW 264.7 cells. All data are expressed as mean ± SD. * *p* < 0.05 compared with LPS treated cells only.

The anti-inflammatory effect of *K. parviflora* was examined by investigating the expression of inflammatory enzymes and their products. The expression pattern of inducible NO synthase (iNOS) and COX-2 was examined by western blotting analysis (Figure 2A). The expression of iNOS induced by lipopolysaccharide (LPS) was drastically decreased depending on the concentration of the *K. parviflora* extracts. COX-2 expression was also reduced at 20 μg/mL of *K. parviflora* extract. Enhanced production of NO by iNOS was proved to trigger the acute and chronic inflammation involved in tissue damage [13]. As shown in Figure 2B, *K. parviflora* extract significantly suppressed the production of NO in murine macrophage-like cell line (RAW 264.7) cells in a concentration-dependent manner. *K. parviflora* extract at 20 μg/mL decreased NO level to that observed in the control. These results showed that the *K. parviflora* extract inhibited the expression of iNOS, which subsequently reduced the production of NO, a key mediator of inflammatory response. In addition, the LPS-induced elevated level of the cytokine TNF-α was significantly reduced by *K. parviflora* extract (Figure 2C).

**Figure 3:**
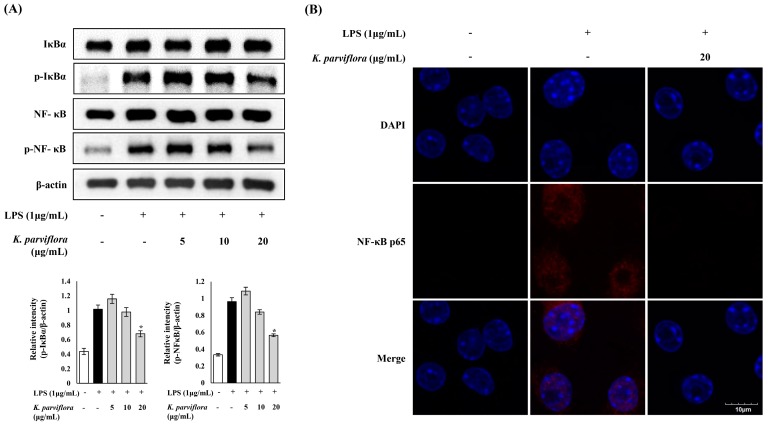
(**A**) Inhibitory effect of *Kaempferia parviflora* extract on the nuclear factor-kappa B (NF-κB) signaling pathway in LPS-stimulated RAW 264.7 cells, examined by western blotting. The expressions of p-IκBα and p-NF-κB were analyzed with ImageJ and normalized against β-actin. * *p* < 0.05 compared with LPS treated cells only; (**B**) effect of *K. parviflora* extract on the translocation of NF-κB p65 in LPS-induced RAW 264.7 cells. Immunofluorescence staining for NF-κB p65 (red) in LPS-exposed RAW 264.7 cells without and with 20 μg/mL *K. parviflora* extract. Nuclei are stained with 4,6-diamidino-2-phenylindole dihydrochloride (DAPI) (blue). *K. parviflora* extract reduced the nuclear translocation and accumulation of NF-κB p65, which was induced by LPS. Scale bar = 10 μm.

Next, the anti-inflammatory effect of *K. parviflora* extract on the nuclear factor-kappa B (NF-κB) signaling pathway was investigated. *K. parviflora* markedly downregulated the expression of phosphorylated inhibitor kappa B-alpha (IκBα) and NF-κB, as examined by western blotting (Figure 3A). These results suggested that the anti-inflammatory activity of *K. parviflora* in LPS-stimulated RAW 264.7 cells might be due to the suppression of the NF-κB signaling pathway. Moreover, *K. parviflora* suppressed the nuclear translocation of p-NF-κB in LPS-stimulated RAW 264.7 cells, as shown in the confocal microscopy data (Figure 3B). Nuclear translocation of p-NF-κB occurred in LPS-stimulated RAW 264.7 cells; however, nuclear translocation and accumulation of p-NF-κB were dramatically reduced by treating the extract. These data show that *K. parviflora* exerted anti-inflammatory effects by modulating NF-κB signaling molecules and p-NF-κB nuclear translocation.

**Figure 4:**
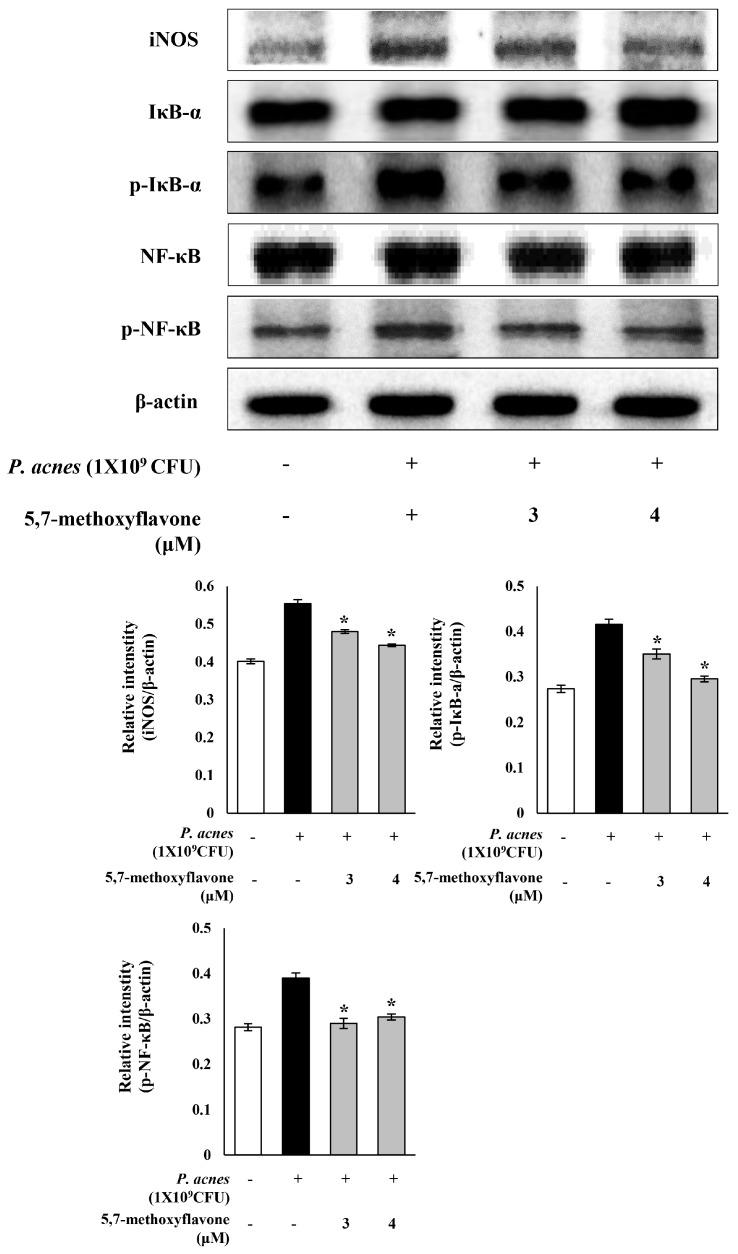
Inhibitory effect of 5,7-methoxyflavone on the *Propionibacterium acnes*-induced expression of inflammatory proteins in *P. acnes*-stimulated human keratinocyte (HaCaT) cells. The expressions of iNOS, p-IκBα and p-NF-κB were analyzed with ImageJ and normalized against β-actin. All data are expressed as mean ± SD. * *p* < 0.05 compared to *P*. *acnes* treated cells only.

Next, we evaluated whether 5,7-methoxyflavone, a major active component of *K. parviflora*, contributes to the anti-inflammatory effect of *K. parviflora* extract and whether the extract is useful to treat *P. acnes*-induced acne vulgaris. The presence of 5,7-dimethoxyflavone in *K. parviflora* extract was identified by UPLC-QTOF-MS in terms of mass and UV peak. The mass of 282 ([M + H]+ *m/z* 283) and a UV λ_max_ at 220, 263, 307 nm characteristic for some flavonoids were found, indicating the presence of 5,7-dimethoxyflavone in our extract. In addition, 5,7-methoxyflavone was applied on *P. acnes*-infected human keratinocyte (HaCaT) cells as a cell-based acne model. The expression of the iNOS enzyme and the NF-κB signaling molecules, IκBα and NF-κB, were investigated by western blotting. As shown in Figure 4, the *P. acnes*-induced expression of iNOS was significantly reduced by 5,7-methoxyflavone. Moreover, phosphorylated IκB-α and NF-κB were notably downregulated by 5,7-methoxyflavone. These results suggested that the anti-inflammatory effect of *K. parviflora* extract might be due to the presence of 5,7-methoxyflavone, and mediated through inhibition of the expression of the iNOS and NF-κB signaling molecules in *P. acnes*-stimulated HaCaT cells. Thus, *K. parviflora* extract might be useful to treat the inflammatory response of acne vulgaris.

### 2.3. Sebostatic Effect of K. parviflora Extracts

**Figure 5:**
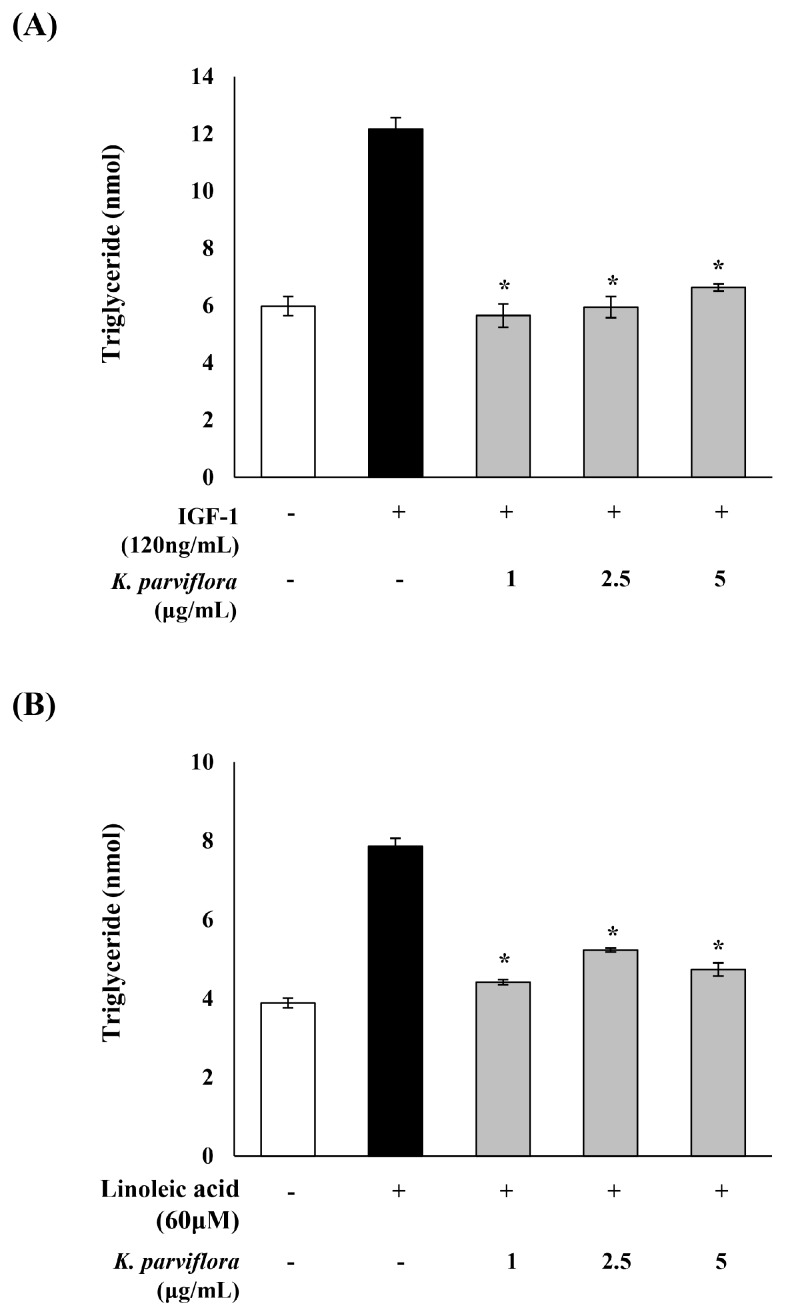
Effects of *Kaempferia parviflora* extract on (**A**) IGF-1- and (**B**) linoleic acid-induced lipogenesis. Triglyceride (TG) levels were analyzed by enzyme-linked immunosorbent assay (ELISA). All data are expressed as mean ± SD. * *p* < 0.05 compared to IGF-1 or linoleic acid treated cells only.

The sebostatic effect of *K. parviflora* extracts on the level of triglyceride, a major lipid in sebum, was examined. IGF-1 and LA were added to sebocytes to promote sebum production (Figure 5). The triglyceride (TG) content in sebocytes, which was doubled by IGF-1 and LA, was significantly reduced by *K. parviflora* extract at 1, 2.5 and 5 µg/mL.

**Figure 6:**
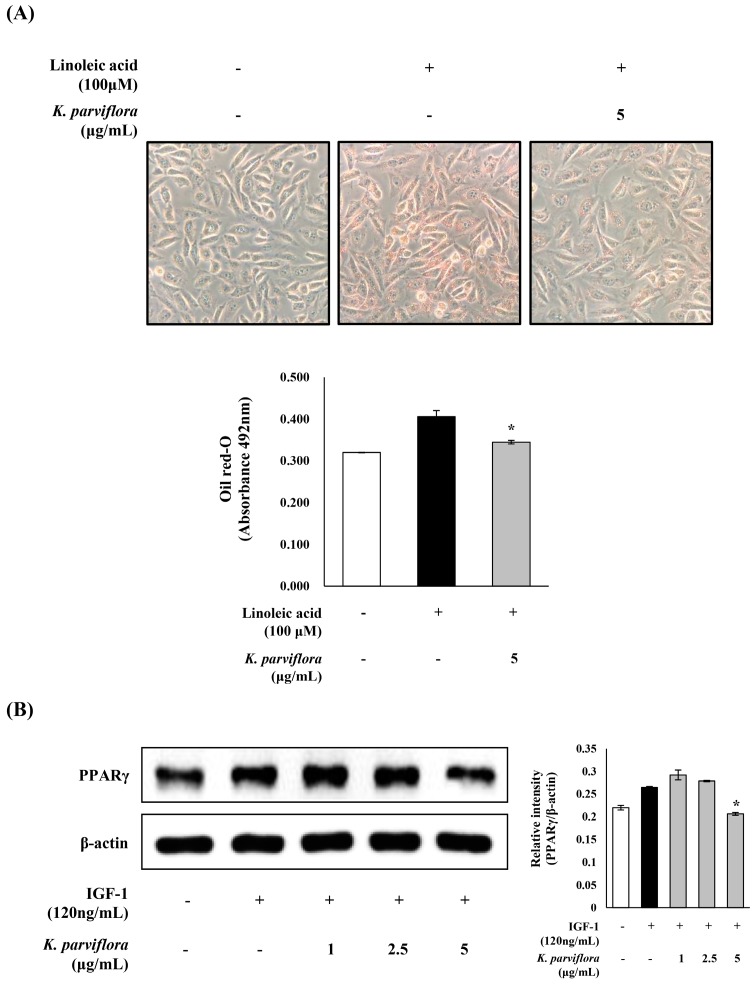
(**A**) Effects of *Kaempferia parviflora* extract on lipid synthesis in sebocytes. The lipid content of sebocytes was detected by Oil red O staining and then measured by ELISA. The sebocytes were shown by microscopy at a magnification of ×100; (**B**) effects of *K. parviflora* extract on the expression of IGF-1-induced peroxisome proliferator-activated receptor gamma (PPAR-γ). The expressions of PPAR-γ was analyzed with ImageJ and normalized against β-actin. All data are expressed as mean ± SD. * *p* < 0.05 compared to IGF-1 or linoleic acid treated cells only.

Moreover, the LA-accumulated intracellular lipid was reduced in sebocytes treated with 5 μg/mL *K. parviflora* extract (Figure 6A), as examined by oil red O staining. The IGF-1-induced expression of peroxisome proliferator-activated receptor gamma (PPAR-γ) in sebocytes was also significantly inhibited by 5 μg/mL *K. parviflora* extract (Figure 6B).

## 3. Discussion

### 3.1. K. parviflora Extract Inhibits the Growth of Skin Bacteria

The growth of bacterial skin flora depends on the condition of the skin environment which they populate. *Propionibacterium* species reside predominately in the sebaceous areas, whereas *Staphylococcus* species typically populate the dry surface of the skin [14,15]. *P. acnes* is involved in an inflammatory response and lipogenesis in acneic skin, contributing to the development and aggravation of acne because it metabolizes sebum into fuel for its growth in the clogged pores of acne lesions [16,17]. *S. aureus* is a bacterium commonly found on the skin surface. However, it can cause many life-threatening infections when it enters the body’s system. In this study, *K. parviflora* extract showed a significant antimicrobial effect against these two causative agents of acne vulgaris. Our results suggested that *K. parviflora* extract might be useful to control skin disorders triggered by these bacteria.

### 3.2. K. parviflora Extract Inhibits Inflammatory Responses

Inflammation has been suggested as a key factor involved in the development and aggravation of acne vulgaris [18], although the exact mechanisms underlying the pathogenesis and subsequent development of acne are not clarified fully. Recently, many efforts have been made to elucidate novel therapeutic targets for acne, including modulators of the signalling molecules in the inflammatory response. On the basis of this information, the inhibitory effect of *K. parviflora* extract on inflammation was investigated in advance by using a general inflammatory model with LPS and RAW 264.7 cells. LPS stimulation in macrophages has been widely known to play a critical role in inflammatory response by releasing pro-inflammatory cytokines, nitric oxide and PGE_2_ [19,20].

Using Western blotting analysis, we showed that the LPS-induced expression of iNOS was drastically decreased by *K. parviflora* extract in a concentration-dependent manner (Figure 2A). Moreover, *K. parviflora* extract, which inhibited the expression of iNOS, subsequently reduced the production of NO (Figure 2B), a key mediator of inflammatory response. In addition, *K. parviflora* extracts also reduced the expression of COX-2 and the production of PGE_2_ to some extent. These results suggested that the extract exerted its anti-inflammatory activity via downregulation of iNOS and COX-2 expression. Anti-inflammatory activities associated with iNOS and COX-2 expression were reported by a variety of herbs [21,22,23] and herbal formulae [24]. COX-2 and iNOS can be induced by many of the same cytokines, and expressed together in inflamed tissues. Specifically, NO produced by iNOS enhances COX-2 activity through peroxynitrite-mediated activation of the peroxidase activity of COX-2 [25].

Next, *K. parviflora* extract modulated NF-κB signaling in LPS-induced RAW 264.7 cells. Activation of NF-κB mainly occurs via IκB kinase-mediated phosphorylation of IκBα. Phosphorylated IκB protein is then ubiquitinated and degraded, separating the inactive NF-κB from IκB, leading to an active state. LPS-activated NF-κB enters the nucleus and binds to DNA to activate the transcription of several genes related to inflammation and cell death. In this study, the expressions of phosphorylated IκBα and NF-κB p65 in RAW 264.7 cells were upregulated in response to LPS stimulation. However, *K. parviflora* extract at 20 μg/mL drastically downregulated the increased expression of phosphorylated IκBα and NF-κB (Figure 3A). Therefore, *K. parviflora* extract exerted an anti-inflammatory effect through modulating the NF-κB pathway. Moreover, the LPS-induced nuclear translocation and the accumulation of NF-κB p65 was also markedly reduced by *K. parviflora* extract (Figure 3B). In addition, the anti-inflammatory effect of *K. parviflora* extract might be related to the presence of 5,7-methoxyflavone. Further, 5,7-methoxyflavone reduced the levels of phosphorylated IκB-α and NF-κB, as well as iNOS, in *P. acnes*-stimulated HaCaT cells, which is a cell-based acne model (Figure 4). In conclusion, *K. parviflora* extracts, which were exerting an anti-inflammatory effect due, most probably, to the presence of 5,7-methoxyflavone, might be effective in controlling the inflammatory acne vulgaris.

### 3.3. The Anti-Lipogenesis Effect of K. parviflora Extract in Sebocytes

Recently, acne was defined as an inflammatory disease primarily triggered by pro-inflammatory sebum lipid fractions [26]. Thus, the relationship between inflammation and lipogenesis might be crucial in fully elucidating acne pathogenesis. Sebum is a mixture of lipids composed mainly of triglycerides, wax esters, squalene, fatty acids, and low amounts of cholesterol. Excess production of sebum is attributable to inflammatory disorders associated with the excessive growth of *P. acnes.* In addition, alterations in sebum lipid composition also play a crucial role in the clinical development and aggravation of acne [27]. The ratio of saturated to unsaturated fatty acids was found to have changed in the sebum of acne patients. In particular, increases in the levels of squalene peroxide and wax esters and the C16:0/C16:1 ratio, as well as decreases in LA and vitamin E contents were found in acne patients [6]. Low-level linoleic acid and sphingolipids have been involved in follicular hyperkeratosis and linked with comedone formation, epidermal barrier dysfunction, and elevated permeability of the comedonal wall for environmental stressors [28].

In particular, an altered proportion of monounsaturated fatty acids associated with desaturation of fatty acids may induce acne onset. Notably, the ratio between Δ6 and Δ9 unsaturated fatty acids has been reported to be a biomarker for sebaceous cell maturation [6,29]. In addition, an acne lesion contains an accumulated level of lipid peroxides, specifically squalene peroxide. A high level lipid peroxide could activate the peroxisome proliferator activated receptors, thereby stimulating lipoxygenase activity and subsequently enhancing the expression of pro-inflammatory cytokines in acne, as well as providing suitable environments for *P. acnes* proliferation [30,31,32].

In this study, the inhibitory effect of *K. parviflora* extract on triglyceride level in sebocytes was examined (Figure 5). IGF-1 and LA, which promote sebaceous lipogenesis and secretion through sebocyte differentiation, were applied to promote sebum production in sebocytes. IGF-1 and LA-induced an increase in triglyceride level in sebocytes which was significantly decreased by treatment with *K. parviflora* extract. Moreover, the inhibitory effect of *K. parviflora* extract on lipid accumulation in sebocytes was also confirmed by Oil red O staining (Figure 6A).

Lipogenesis in sebaceous follicles is stimulated by upregulation of PPAR-γ. Thus, specific PPAR-γ antagonists might be regarded as candidates for anti-acne agents. In this study, the IGF-1-induced expression of PPAR-γ in sebocytes was diminished by *K. parviflora* extract, postulating that *K. parviflora* extract could be developed as an anti-acne agent with a sebostatic effect associated with the inactivation of PPAR-γ. Currently, sebostatic agents inhibiting sebum lipogenesis, such as DRM01, are regarded as promising candidates for anti-acne agents [33,34,35].

**Figure 7:**
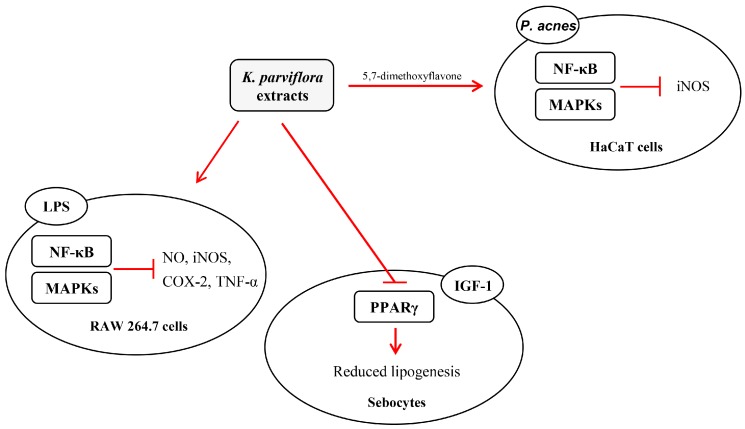
Proposed underlying mechanism of the anti-acne effect of *Kaempferia parviflora.* The t-bar denotes an inhibitory effect.

In this study, *K. parviflora* extract effectively suppressed the growth of acne-causing skin bacteria. Moreover, the extract downregulated inflammatory responses by regulating the iNOS and NF-κB signaling, and lipogenesis by modulating lipogenesis and PPAR-γ expression (Figure 7). Thus, *K. parviflora* extract might be used as a potential anti-acne agent targeting inflammation and lipogenesis triggered by acne-causing bacteria.

## 4. Materials and Methods

### 4.1. Preparation of K. parviflora Extracts in Sebocytes

Rhizomes of *K. parviflora*, collected in Bangkok, Thailand, were supplied by Biocm Co., Ltd. (Asan, Korea), and used for the experiment. The dried rhizomes were ground and then soaked in 95% ethanol for 24 h at room temperature. *K. parviflora* filtrate was concentrated under reduced pressure using a rotary evaporator to obtain *K. parviflora* extracts with a yield of 15.3%. The presence of 5,7-dimethoxyflavone in the extract was identified by UPLC-QTOF-MS in terms of mass and UV peak.

### 4.2. Microbial Cultivation

*Propionibacterium acnes* (KCTC 3314) and *Staphylococcus aureus* subsp. *aureus* (KCTC 1927) were obtained from the Korean Culture Center of Microorganisms (Seoul, Korea). *P. acnes* was grown anaerobically in reinforced clostridial medium (RCM) broth at 37 °C for 72 h. *S. aureus* was grown aerobically in Luria-Bertani (LB) at 37 °C for 12–24 h [36].

### 4.3. Bacterial Inactivation by K. parviflora Extract

Bacterial culture was standardized using a #0.5 McFarland standard solution according to the recommendations of the clinical laboratory standard institute [37]. *S. aureus* and *P. acnes* were cultured and treated with each concentration of *K. parviflora* extract. The broth microdilution method using a 96-well microtiter plate was used to measure the antimicrobial effect of *K. parviflora* extract [38,39]. Bacterial suspensions were diluted to 1.5 × 10^5^ CFU/mL, and *K. parviflora* extract was added. Incubation proceeded at 37 °C under anaerobic conditions for 72 h or aerobic conditions for 24 h. The absorbance of the bacterial suspensions at 620 nm was measured to estimate bacterial growth inhibition.

### 4.4. Cell Culture

A cell-based acne model was constructed according to our previous reports [20,36,40]. The RAW 264.7 and HaCaT were obtained from the Global Bioresource Centre (ATCC, Manassas, USA) and incubated in complete Dulbecco’s Modified Eagle’s Medium (DMEM; Hyclone, Logan, UT, USA) containing 100 U/mL penicillin, 100 μg/mL streptomycin, and 10% fetal bovine serum (FBS) at 37 °C. Primary human sebocytes were obtained from Celprogen (San Pedro, CA, USA) and maintained in Human Sebocyte Complete Growth Media from the same vendor. Cells were seeded and incubated overnight prior to treatment with *K. parviflora* extract [32,40]. For the stimulation experiment, HaCaT cells were incubated with heat-killed *P. acnes* adjusted at the appropriate concentration in serum-free media for 24 h at 37 °C in 5% CO_2_. After stimulation, HaCaT cells were treated with or without 5,7-methoxyflavone (Sigma-Aldrich Corp., St. Louis, MO, USA) for 48 h at 37 °C in 5% CO_2_ [41].

### 4.5. Nitrite Determination

RAW 264.7 cells were seeded and incubated overnight prior to treatment with *K. parviflora* extract. The cells were treated with various concentrations of *K. parviflora* extract for 18 h with or without subsequent exposure to 1 μg/mL LPS at 37 °C in an atmosphere of 5% CO_2_ in the dark. NO level was determined by measuring the concentration of the end product, nitrite, using the Griess assay. Briefly, culture supernatant (100 mL) was mixed with 150 mL of Griess solution (1:1 mixture (*v*/*v*) of 1% sulfanilamide and 0.1% *N*-(naphthyl) ethylenediamine dihydrochloride in 5% H_3_PO_4_) in 96-well plates at room temperature for 5 min. Absorbance at 570 nm was measured by a microplate reader, and nitrite concentration in the cultures was calculated using a standard curve of sodium nitrite [32,37].

### 4.6. Cytokine Measurement

RAW 264.7 cells were seeded into a 24-well plate at a density of 2.5 × 10^5^ cells/well and incubated overnight prior to the treatments. Cells were pretreated with 0, 5, 10, or 20 μg/mL *K. parviflora* extract for 2 h. Afterward, 1 μg/mL LPS was added to each well and the cells were incubated for 12 h. The supernatant was transferred to an ELISA plate and TNF-α level in the culture medium was determined using a commercial kit (Mouse TNF-α ELISA kit, BD, Franklin lakes, USA) according to the manufacturer’s instruction [42].

### 4.7. Western Blotting Analysis

Proteins were separated by 10% SDS-PAGE and then transferred onto polyvinylidene fluoride membranes (Bio-Rad Laboratories, Hercules, CA, USA). The membranes were incubated overnight at 4 °C with primary antibodies (iNOS, IκBα, p-IκBα, NF-κB p65, p-NF-κB p65 (Cell Signaling Technology, Danvers, USA), COX-2, PPAR-γ, and β-actin (Santa Cruz Biotechnology, Dallas, USA)) which were diluted following the manufacturers’ recommendations. The membranes were then washed in mixture of tris-buffered saline and tween 20 (TBST) and incubated with the appropriate horseradish peroxidase (HRP)-conjugated secondary antibody (1:5000) at room temperature for 1 h. Protein bands were visualized using a Sensi-Q 2000 (Lugen, Korea). The intensity of the bands was analyzed using the ImageJ software (1.50i) and normalized against that of β-actin [41,43].

### 4.8. Confocal Microscope Analysis

LPS-induced inflammation in RAW 264.7 cells was examined as follows. The cells were treated with *K. parviflora* extract, fixed with 4% paraformaldehyde in PBS for 20 min, and permeabilized with 0.5% Triton X-100 for 15 min. After 1 h of incubation with a blocking buffer (5% BSA in PBS), the cells were incubated with primary antibodies (rabbit monoclonal antibodies against NF-κB p65; 1:100) (Cell Signaling Technology, Inc., Danvers, MA, USA) in 0.5% BSA overnight at 4 °C. The cells were washed three times with PBS for 10 min and stained for another 1 h with goat anti-rabbit IgG Texas red (1:1000) (Santa Cruz Biotechnology, Inc., Dallas, USA). Nuclei were counterstained with 4,6-diamidino-2-phenylindole dihydrochloride (DAPI) (Bio-Rad, Hercules, USA). The prepared cells were then observed under a fluorescent microscope and images were recorded [41].

### 4.9. Sebocyte Culture and Triglyceride Assay

Sebocytes were seeded into a 100 mm cell culture plate at a density of 2 × 10^6^ cells and incubated overnight prior to the treatments. Cells were pretreated with 0, 5, 10 or 20 μg/mL *K. parviflora* extract for 2 h. Afterward, 120 ng/mL IGF-1 or 60 μM LA was added to each plate and the cells were incubated for 12 h. The cells were washed with PBS and 5% NP-40 in PBS was added to lyse the cells. Next, the cells were assayed using a Triglyceride Quantification Kit (Abcam, Cambridge, UK) according to the manufacturer’s instructions [44]. 

### 4.10. Oil Red O Staining

Oil red O stock solution was prepared using isopropyl alcohol diluted in distilled water at a ratio of 3:2. Next, cells were fixed with formalin for 10 min at room temperature. After washing, the cells were stained with Oil red O solution for 1 h at room temperature and washed with 60% isopropyl alcohol. The stained cells were photographed using a CCD camera (IMT cam CCD Pro2; IMT i-Solution Inc., Vancouver, BC, Canada). Cell morphology was visualized by microscopy. Stained lipid droplet in the cells was quantified by eluting the cells in isopropanol and placing 200 μL aliquots of the elution onto 96-well plates. The optical density of each well was determined using an ELISA reader (Sunrise, Tecan, Switzerland) at 492 nm [45].

### 4.11. Statistical Analysis 

All data are presented as mean ± SD of triplicate experiments. The one-way analysis of variance (ANOVA) and Duncan multiple-comparison test were utilized to determine statistical differences among the groups. *p*-values < 0.05 were considered statistically significant [32].
